# DeepQuality improves infant retinopathy screening

**DOI:** 10.1038/s41746-023-00943-3

**Published:** 2023-10-16

**Authors:** Longhui Li, Duoru Lin, Zhenzhe Lin, Mingyuan Li, Zhangkai Lian, Lanqin Zhao, Xiaohang Wu, Lixue Liu, Jiali Liu, Xiaoyue Wei, Mingjie Luo, Danqi Zeng, Anqi Yan, Wai Cheng Iao, Yuanjun Shang, Fabao Xu, Wei Xiang, Muchen He, Zhe Fu, Xueyu Wang, Yaru Deng, Xinyan Fan, Zhijun Ye, Meirong Wei, Jianping Zhang, Baohai Liu, Jianqiao Li, Xiaoyan Ding, Haotian Lin

**Affiliations:** 1 State Key Laboratory of Ophthalmology, Zhongshan Ophthalmic Center, Sun Yat-sen University, Guangdong Provincial Key Laboratory of Ophthalmology and Visual Science, Guangdong Provincial Clinical Research Center for Ocular Diseases, Guangzhou, China; 2 Department of Ophthalmology, Qilu Hospital, Shandong University, Jinan, Shandong China; 3 Department of Clinical Laboratory Medicine, Guangdong Provincial People’s Hospital (Guangdong Academy of Medical Sciences), Southern Medical University, Guangzhou, China; 4 Zhongshan School of Medicine, Sun Yat-sen University, Guangzhou, Guangdong China; 5 Department of Ophthalmology, Maternal and Children’s Hospital, Liuzhou, Guangxi China; 6 Department of Ophthalmology, Maternal and Children’s Hospital, Linyi, Shandong China; 7 Hainan Eye Hospital and Key Laboratory of Ophthalmology, Zhongshan Ophthalmic Center, Sun Yat-sen University, Haikou, Hainan China; 8 Center for Precision Medicine and Department of Genetics and Biomedical Informatics, Zhongshan School of Medicine, Sun Yat-sen University, Guangzhou, Guangdong China

**Keywords:** Translational research, Eye diseases

## Abstract

Image quality variation is a prominent cause of performance degradation for intelligent disease diagnostic models in clinical applications. Image quality issues are particularly prominent in infantile fundus photography due to poor patient cooperation, which poses a high risk of misdiagnosis. Here, we developed a deep learning-based image quality assessment and enhancement system (DeepQuality) for infantile fundus images to improve infant retinopathy screening. DeepQuality can accurately detect various quality defects concerning integrity, illumination, and clarity with area under the curve (AUC) values ranging from 0.933 to 0.995. It can also comprehensively score the overall quality of each fundus photograph. By analyzing 2,015,758 infantile fundus photographs from real-world settings using DeepQuality, we found that 58.3% of them had varying degrees of quality defects, and large variations were observed among different regions and categories of hospitals. Additionally, DeepQuality provides quality enhancement based on the results of quality assessment. After quality enhancement, the performance of retinopathy of prematurity (ROP) diagnosis of clinicians was significantly improved. Moreover, the integration of DeepQuality and AI diagnostic models can effectively improve the model performance for detecting ROP. This study may be an important reference for the future development of other image-based intelligent disease screening systems.

## Introduction

Expectations for artificial intelligence (AI) to transform conventional healthcare modes across various specialties have been growing in recent years^1,2^. Within the field of AI, image-based AI systems are the closest to clinical implementation due to the advancement of deep learning techniques^3^. In routine ophthalmic clinical practice, fundus photography is the most common examination modality, and automated identification of various fundus diseases based on fundus images was also the earliest focus of medical AI studies^4,5^. IDx-DR^6^, an automated screening software for diabetic retinopathy (DR), is the first certificated AI diagnostic software based on fundus photography in the world^7^. Numerous AI diagnostic models for other fundus diseases based on fundus photography have also been developed and reported to have excellent performance^8–10^. However, most AI-based models show a significant decline in performance when using real-world data, thus hindering their clinical applications. The dataset shift caused by image quality variation has been recognized as one of the primary reasons for the performance degradation of the models in clinical practice^11^. Most previous studies used a selection of good quality images to develop and evaluate the models. Nevertheless, a substantial portion of the images in clinical practice have quality defects.

Image quality issues are particularly prominent in infantile fundus photography due to poor patient cooperation. It has been reported that 49.4% of fundus photographs in infants in clinical practice have quality defects^12^, which is much higher than the proportion of 11.7% in adults^6^ and poses a higher risk of misdiagnosis. Retinopathy of prematurity (ROP) is the leading cause of preventable infantile blindness^13^ and is primarily diagnosed based on disease characteristics in the fundus. To improve the efficiency of ROP screening and promote ROP screening on a broad scale, several AI models based on fundus photographs for ROP screening have been developed^14,15^. However, the prominent quality issues in real-world settings significantly degrade model performance and thus limit them from large-scale clinical applications. To date, there are no licensed AI disease screening technologies based on infantile fundus photography, likely due to unstable image quality^16^.

To address the image quality issues, the initial approach was to manually classify images. This approach is subjective and time-consuming and is not suitable for high-throughput data scenarios, such as disease screening. Several automated fundus image quality assessment models have also been previously proposed^17–21^. However, there are still several critical limitations that must be addressed. First, these models mostly divided fundus images into acceptable or unacceptable quality and could not pinpoint the specific causes of poor quality, which prevented photographers from making adjustments accordingly. Moreover, low-quality images were discarded in most previous studies, but infantile fundus images are difficult to capture, and simply discarding these images would result in a great waste of data resources.

The aim of this study is to develop DeepQuality, a deep learning-based infantile fundus image quality assessment and enhancement system. We showed that DeepQuality provides systematic quality assessment in terms of integrity, illumination, and clarity. It can also comprehensively score the image quality of each fundus photograph. Moreover, DeepQuality can provides quality enhancement based on the results of quality assessment. DeepQuality significantly improve ROP diagnostic performance of both clinicians and AI models. This system may improve infant retinopathy screening in clinical practice and provide a reference for the development of other image-based disease diagnostic systems.

## Results

### Data distribution in the workflow

A total of 2,056,260 infantile fundus images were enrolled for DeepQuality development. Among them, 32,112 fundus images were labeled according to quality annotation criteria and used to develop and evaluate the quality assessment module; 2,015,748 fundus images were enrolled to investigate the real-world image quality distribution; and 8400 fundus images were labeled according to quality grading and characteristics of ROP, then used to develop the quality scoring module and perform the ROP diagnostic test after quality enhancement.

### Performance of the multidimensional quality assessment module

A total of 32,112 fundus images from 4028 infants were from routine infantile fundus screening cohorts from four centers. Specifically, 5986 posterior fundus images and 26,766 peripheral fundus images were used to develop and evaluate the multidimensional quality classification module. This module can identify the location (posterior or peripheral) of fundus images and detect quality defects in three dimensions, namely, illumination, clarity, and integrity. Corresponding examples of different image quality defects are shown in Supplementary Fig. 1. The clinical characteristics of the subjects and the distribution of images at each quality aspect are summarized in Supplementary Table 1 and Table 1.

**Table 1:** Characteristics of the development, internal test, and external test datasets.

	Development dataset (*N* = 15,998)		Internal test dataset (*N* = 4001)		External test dataset (*N* = 12,753)						Inter-grader consistency
					LZH		LYH		QLH		
	GQ	PQ	GQ	PQ	GQ	PQ	GQ	PQ	GQ	PQ	
Posterior (*N* = 5986)											
Illumination, macula	2187	584	542	151	761	216	585	247	582	131	0.962
Illumination, optic disc	2599	172	650	43	863	114	711	121	671	42	0.945
Illumination, remaining retina	2488	283	613	80	928	49	763	69	688	25	0.943
Clarity, macula	2265	506	572	121	734	243	582	250	548	165	0.868
Clarity, optic disc	2411	360	597	96	743	234	602	230	522	191	0.870
Clarity, remaining retina	2168	603	525	168	646	331	589	243	475	238	0.875
Peripheral (*N* = 26,766)											
Integrity, whole retina	10,425	2802	2607	701	2785	1066	2281	791	2607	701	0.970
Illumination, whole retina	11,226	2001	2881	427	3311	540	2809	263	2881	427	0.960
Clarity, whole retina	8186	5041	2047	1261	1870	1981	1592	1480	2047	1261	0.874

*N* number, *w* week, *g* gram, *GQ* good quality, *PQ* poor quality.

With the internal test dataset, DeepQuality achieved an AUC of 0.974 (95% CI: 0.969, 0.979) in distinguishing image location (posterior or peripheral). For posterior fundus images, DeepQuality achieved AUCs of 0.908–0.940 and 0.899–0.955 in distinguishing poor-quality images concerning illumination and clarity. For peripheral fundus images, DeepQuality achieved AUCs of 0.957 (0.951, 0.964), 0.957 (0.950, 0.964), and 0.927 (0.918, 0.935) in distinguishing poor-quality images concerning illumination, clarity, and integrity, respectively. The detailed performance of the internal test is summarized in Supplementary Table 2 and Supplementary Fig. 2.

**Fig. 1:**
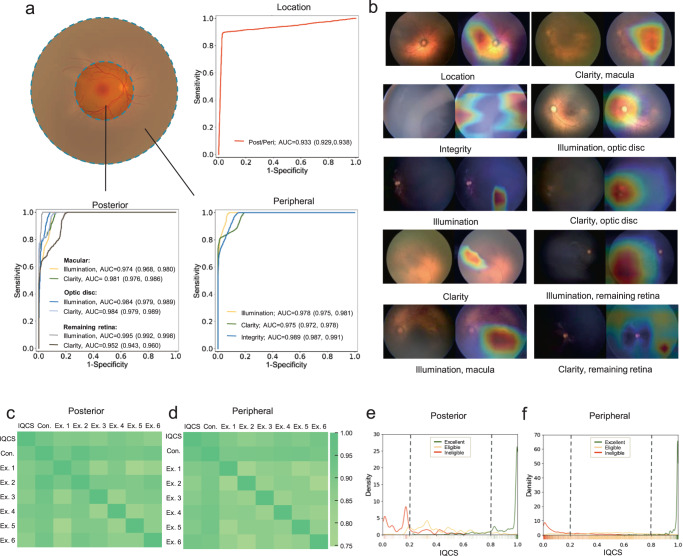
Performance of the quality assessment module. **a** In the external test dataset, the assessment module achieved an AUC of 0.933 for distinguishing the location of fundus images. For posterior images, the assessment module achieved AUCs of 0.952–0.995 for detecting various quality defects. For peripheral images, the assessment module achieved AUCs of 0.975–0.989 for detecting various quality defects. **b** For each of the ten models in the quality assessment module, the original images (left) and corresponding heatmaps (right) are shown. The heatmaps show that the models are focused precisely on the exact regions with quality defects. The correlation matrix shows the Spearman’s rank correlation coefficients between the IQCS ranking, individual experts’ ranking, and consensus ranking in posterior images (**c**) and peripheral images (**d**). **e**, **f** Distributions of IQCS in infantile fundus images with different quality grades. **e** For posterior images, the results are shown for 589 excellent quality images, 446 eligible quality images, and 110 ineligible images. **f** For peripheral images, the results are shown for 573 excellent quality images, 5776 eligible quality images, and 1015 ineligible images. The dashed lines represent the hypothetical thresholds. Post posterior, Peri peripheral, IQCS image quality comprehensive score, Con. consensus, Ex. experts.

In the overall external test dataset including data from three centers, DeepQuality achieved an AUC of 0.933 (0.929, 0.938) in distinguishing image locations (Table 2 and Fig. 1a). For detecting poor illumination in posterior fundus images, the AUCs of DeepQuality were 0.974 (0.968, 0.980), 0.984 (0.979, 0.989), and 0.995 (0.992, 0.998) for the macular area, optic disc, and remaining retina, respectively. For detecting poor clarity in posterior fundus images, the AUCs of DeepQuality were 0.981 (0.976, 0.986), 0.984 (0.979, 0.989), and 0.952 (0.943, 0.960) for the macular area, optic disc, and remaining retina, respectively. For peripheral fundus images, the AUCs of DeepQuality were 0.978 (0.975, 0.981), 0.989 (0.987, 0.991), and 0.975 (0.972, 0.978) for detecting poor integrity, poor illumination, and poor clarity, respectively. The detailed performance information of DeepQuality in the LZH, LYH, and QLH datasets is presented in Supplementary Tables 3–5 and Supplementary Figs. 3–5.

**Table 2:** Model performance in the external tests.

	Model	Sensitivity (95% CI)	Specificity (95% CI)	Accuracy (95% CI)	AUC (95% CI)
Location		0.885 (0.879, 0.891)	0.972 (0.969, 0.975)	0.954 (0.950, 0.958)	0.933 (0.929, 0.938)
Posterior	Illumination, macula	0.823 (0.808, 0.838)	0.926 (0.916, 0.936)	0.902 (0.890, 0.914)	0.974 (0.968, 0.980)
	Illumination, optic disc	0.866 (0.853, 0.879)	0.953 (0.945, 0.961)	0.943 (0.934, 0.952)	0.984 (0.979, 0.989)
	Illumination, remaining retina	0.853 (0.839, 0.867)	0.984 (0.979, 0.989)	0.977 (0.971, 0.983)	0.995 (0.992, 0.998)
	Clarity, macula	0.881 (0.868, 0.894)	0.914 (0.903, 0.925)	0.905 (0.894, 0.916)	0.981 (0.976, 0.986)
	Clarity, optic disc	0.835 (0.821, 0.850)	0.834 (0.820, 0.849)	0.908 (0.897, 0.919)	0.984 (0.979, 0.989)
	Clarity, remaining retina	0.821 (0.806, 0.836)	0.845 (0.831, 0.859)	0.837 (0.823, 0.851)	0.952 (0.943, 0.960)
Peripheral	Integrity	0.878 (0.872, 0.885)	0.918 (0.913, 0.924)	0.908 (0.902, 0.914)	0.978 (0.975, 0.981)
	Illumination	0.921 (0.916, 0.926)	0.945 (0.940, 0.950)	0.942 (0.937, 0.947)	0.989 (0.987, 0.991)
	Clarity	0.879 (0.873, 0.886)	0.869 (0.862, 0.876)	0.874 (0.867, 0.881)	0.975 (0.972, 0.978)

*CI* confidence interval, *Location* posterior or peripheral, *AUC* area under the receiver operating characteristic curve.

Heatmaps were used to visualize the regions with the greatest contribution to each model’s prediction. Typical heatmap examples for each model are presented in Fig. 1b. The results showed that heatmaps for each model accurately specified the quality defects corresponding to poor integrity, poor illumination, and poor clarity.

### Performance of the quality scoring module

IQCS was calculated based on a linear weighting of the probabilities of poor illumination, poor clarity, and poor integrity output by the quality classification module. There were different formulas for the IQCS of posterior images and peripheral images. We evaluated the quality assessment capability of the IQCS through a quality rank test. As shown in Fig. 1c, for posterior fundus images, the Spearman’s rank correlation coefficients between the ranks assigned by IQCS and experts ranged from 0.828 to 0.924. As shown in Fig. 1d, for peripheral fundus images, the Spearman’s rank correlation coefficients between the ranks assigned by the IQCS and the experts ranged from 0.829 to 0.957. These results suggested that there was very high agreement on the relative image quality ranking between the IQCS and experts.

To investigate the appropriate threshold for IQCS in ROP screening scenarios, a dataset consisting of fundus images from the routine ROP screening cohorts from ZOC and QLH was constructed. After quality grading, a total of 1162 excellent quality images, 6223 eligible quality images, and 1015 ineligible quality images were included and analyzed by the quality quantification module (detailed information is presented in Supplementary Tables 6 and 7). The density distribution curve (Fig. 1e, f) shows that the IQCS of images with excellent quality, eligible quality, and ineligible quality are concentrated in the 0.8–1.0, 0.2–0.8, and 0–0.2 ranges, respectively. After grid search, the optimal thresholds of IQCS were set as 0.2 and 0.8, which could distinguish excellent quality, eligible quality, and ineligible quality images with accuracies of 0.842 and 0.858 for posterior and peripheral fundus images, respectively. Further information on the grid search is shown in Supplementary Table 8.

### Large-scale quality assessments for real-world infantile fundus images

The quality analysis of 2,015,748 infant fundus images showed that 39.12% of the images had poor integrity, 35.17% had poor illumination, and 39.95% had poor clarity (Supplementary Table 9). The IQCS mean and standard deviation were 0.345 ± 0.312. Moreover, infant fundus image sets from ophthalmic hospitals had lower proportions of poor integrity (21.2% vs. 45.6%), poor illumination (16.0% vs. 35.2%), and poor clarity (24.1% vs. 33.8%) images than those from maternity and children’s hospitals (MCHs). There were also significant variations in the proportion of quality defects between MCHs in different regions, which ranged from 40.52 to 46.67%. The results of the quality scoring module analysis (Supplementary Table 10) showed that the IQCS distributions varied significantly between ophthalmic and nonophthalmic hospitals. The proportions of infant fundus photographs with IQCS greater than 0.8 in ophthalmic hospitals were 27.8 to 66.4% higher than those from MCH 1 and MCH 2. The IQCS also varies greatly between MCHs in different regions. The IQCSs of the ophthalmic hospital, MCH 1, and MCH 2 were 0.768 ± 0.285, 0.597 ± 0.311, and 0.357 ± 0.294, respectively. The AUCs ranged from 0.857 to 0.947 in the randomized inspection of 2000 fundus images extracted from large-scale real-world dataset (Supplementary Table 11).

### Quality enhancement module for ROP diagnosis improvement

**Fig. 2:**
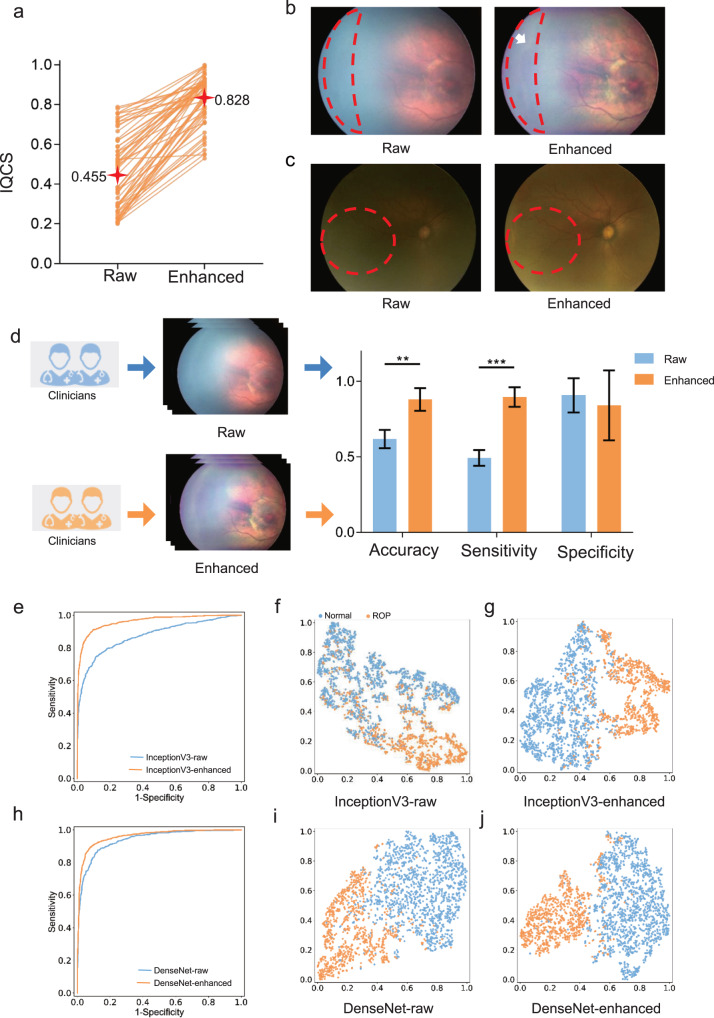
Performance of the quality enhancement module. **a** Comparison of IQCS in infantile fundus images before and after quality enhancement. Each line represents the change in IQCS before and after enhancement of one fundus image. **b**, **c** Typical examples of raw fundus images and the corresponding enhanced fundus images for ROP. Compared to the raw image, the corresponding enhanced image demonstrates better visibility of retinal lesions and vessels. **b** White arrows indicate peripheral retinal ridge-like elevation lesions, which suggest a stage 2 ROP lesion. **c** Red dashed circle indicates that the retinal vessels were tortuous and dilated, which suggests plus-disease of ROP. **d** Clinicians were assigned to diagnose ROP according to 100 raw images and corresponding enhanced images. The accuracy, sensitivity and specificity were compared. The *p* value was calculated using the independent two-sample *t*-test. The error bars represent the standard deviation of the clinicians in the corresponding groups. The performance of ROP diagnosis models developed and evaluated by raw images and corresponding enhanced images using InceptionV3 (**e**) and DenseNet (**h**) were compared. t-Distributed stochastic neighbor embedding visualization of features extracted from an intermediate layer of trained models for ROP diagnosis using InceptionV3 (**f**, **g**) and DenseNet (**i**, **j**) architectures. This visualization demonstrates the capacity of different models to distinguish ROP images and normal images. Orange points represent normal images, and blue points represent ROP images. There was greater intergroup distance and lower intragroup distance in models with the quality enhancement module, which indicated greater performance for ROP diagnosis. ROP retinopathy of prematurity. **p < 0.01; ***p < 0.001.

After excluding ineligible images, a total of 2201 ROP images and 5184 normal images were included for ROP diagnosis tests (Supplementary Table 12). For the test with clinicians, 70 ROP images and 30 normal images were randomly selected. In this dataset of raw images, 59 images had an IQCS ranging from 0.2 to 0.8, and 41 images had an IQCS ≥ 0.8. After quality enhancement, the overall IQCS was improved from 0.455 ± 0.189 to 0.828 ± 0.124 (Fig. 2a), representing an 82% increase and a narrower SD. The ridge-like elevation lesions and plus-disease of ROP were much more visible after quality enhancement (Fig. 2b, c). Clinicians in the enhanced image group exhibited higher sensitivity (0.896 ± 0.057 vs. 0.493 ± 0.046, an increase of 81.6%) and accuracy (0.880 ± 0.065 vs. 0.618 ± 0.053, an increase of 42.4%) for ROP diagnosis than those in the raw image group (Fig. 2d).

For the test with AI diagnostic models, the distribution of retinal images in ROP classification is summarized in Supplementary Table 12. The performance of deep learning-based ROP diagnostic models using InceptionV3 and DenseNet is demonstrated in Table 3. The results showed that the ROP diagnostic model trained with enhanced images performed better than the corresponding model trained with raw images. In models with the InceptionV3 architecture, significant improvement was found in terms of sensitivity (0.508 vs. 0.670, *p* < 0.0001), specificity (0.966 vs. 0.991, *p* < 0.0001), accuracy (0.827 vs. 0.875, *p* < 0.0001) and AUC (0.845 vs. 0.962, *p* < 0.0001) after quality enhancement (Fig. 2e). In models with the DenseNet architecture, sensitivity (0.436 vs. 0.540, *p* < 0.0001), accuracy (0.791 vs. 0.827, *p* = 0.0022), and AUC (0.939 vs. 0.961, *p* < 0.0001) were significantly improved after quality enhancement (Fig. 2h). In addition, t-distributed stochastic neighbor embedding (t-SNE) was employed to visualize the features learned by the DL models (Fig. 2f, g, i, j). The results of t-SNE showed that the point clusters of normal and ROP images had greater intergroup distances and lower intragroup distances after quality enhancement.

**Table 3:** Model performance comparisons for ROP detection before and after image quality enhancement.

Model architecture	Metrics (95% CI)	Model using raw images	Model using enhanced images	*p* value
InceptionV3	Sensitivity	0.508 (0.560, 0.600)	0.670 (0.651, 0.689)	<0.0001*
	Specificity	0.966 (0.959, 0.974)	0991 (0.987, 0.995)	<0.0001*
	Accuracy	0.827 (0.812, 0.842)	0.875 (0.862, 0.889)	<0.0001*
	AUC	0.875 (0.861, 0.889)	0.962 (0.954, 0.970)	<0.0001*
DenseNet	Sensitivity	0.436 (0.416, 0.456)	0.540 (0.520, 0.561)	<0.0001*
	Specificity	0.992 (0.988, 0.996)	0.989 (0.985, 0.993)	0.5689
	Accuracy	0.791 (0.775, 0.808)	0.827 (0.812, 0.843)	0.0022*
	AUC	0.939 (0.929, 0.949)	0.961 (0.953, 0.969)	<0.0001*

*p* values for sensitivity, specificity, and accuracy were calculated using a two-proportion *z* test. The *p* value for AUC was calculated using the DeLong test.

*AUC* area under the receiver operating characteristic curve, *ROP* retinopathy of prematurity.

*p < 0.05.

## Discussion

DeepQuality, the first deep learning-based system for systematic quality assessment and quality enhancement of infant fundus images, was developed in this study. The quality assessment module of DeepQuality could classify fundus image quality in terms of illumination, clarity, and integrity. In both the internal test and multiple external tests, nearly all of the AUCs of quality assessment were greater than 0.9, demonstrating the precision and generalizability of the system. DeepQuality could also comprehensively score overall image quality based on IQCS. The rankings of overall quality by IQCS and retinal experts were highly correlated (Spearman’s rank correlation >0.8). With the assistance of DeepQuality, we verified that 58.3% of infant fundus images had quality issues in a real-world dataset containing over two million images. Significant discrepancies in image quality were observed among different hospitals. Given such a large proportion of quality issues, DeepQuality also provided quality enhancement based on the results of quality assessment. In the ROP diagnosis tests, both clinicians and AI diagnostic models performed better after quality enhancement.

Several previous studies have constructed deep learning-based models for automated fundus image quality assessment. Mahapatra et al. developed the first retinal image quality assessment system based on deep learning with an accuracy of 0.98 in distinguishing gradable and ungradable fundus images^18^. Li et al. constructed a deep learning-based image filtering system (DLIFS) for detecting poor-quality ultrawide-field fundus images with AUCs greater than 0.99 in three external datasets^22^. Compared with these studies, DeepQuality has several advantages. First, DeepQuality provides a multidimensional quality assessment that includes illumination, clarity, and integrity, making it more specific and clinically relevant. Therefore, operators can know the specific cause of poor quality and make adjustments accordingly. Second, most previous studies developed image quality assessment systems for fundus images in adults^23,24^. Few prior studies have focused on deep learning systems for the systematic assessment of infantile fundus image quality. Third, our system can evaluate both posterior and peripheral fundus images, while previous studies only focused on posterior fundus images. The mode proposed in the current study is more ideal for infantile fundus screening scenarios, given that many significant features of infantile fundus disease appear in the peripheral retina and that peripheral fundus images are more likely to suffer from quality issues.

Analysis of real-world infantile fundus photographs with DeepQuality showed that more than half of the images had quality issues. Moreover, there are significant differences in the proportion and causes of poor image quality among hospitals of different categories and regions. These variations may be due to different photography proficiency, equipment conditions, pupil dilatation, and infant cooperation. Poor quality and variation can lead to dataset shifts among hospitals, which in turn could affect the performance of AI diagnostic models. DeepQuality could provide quality enhancements for images with poor illumination and poor clarity with digital technology. After quality enhancement, images exhibited a higher IQCS with a narrower standard deviation. This indicates a significant improvement and decreased variation in image quality.

To investigate whether image quality enhancement could benefit the diagnosis of infantile fundus disease, comparisons of ROP diagnosis were conducted. The results showed that ophthalmologists present better ROP diagnostic performance after quality enhancement, with an 81.6% increase in sensitivity and a 42.4% increase in accuracy. Moreover, the AI diagnostic models trained with enhanced images exhibited better performance than those trained with the corresponding raw images (with AUCs of 0.96 vs. 0.88). These results indicated that DeepQuality has great potential to improve the performance of ROP diagnostic models in real-world settings. According to the clinical consensus^25^, the diagnosis of ROP is based on the degree of dilatation and tortuosity of the retinal vessels, as well as the appearance of structures at the vascular-avascular juncture. Quality enhancement makes the retinal ridge and tortuous vessels of ROP more visible and easier to identify. A prepositive image quality assessment and enhancement system is necessary to ensure the performance of AI diagnostic models in infant retinopathy screening.

There are some limitations in this study. First, quality enhancement does not solve all quality defects. In this study, illumination and clarity defects can be enhanced by digital methods, while integrity issues still need to be addressed by rephotographing the fundus. Moreover, only images with mild and moderate quality issues (with IQCS ranging from 0.2 to 0.8) can be improved, and images with severe quality defects (IQCS < 0.2) should be discarded. Second, we developed a quality assessment and enhancement system and proved its utility in retrospective datasets. However, the effectiveness of DeepQuality in real-world settings still needs to be prospectively validated. Third, DeepQuality is theoretically useful for other infantile fundus diseases, such as choroidal coloboma, retinoblastoma, and optic nerve developmental abnormalities, but only ROP was investigated in this study. The assistance of DeepQuality to other infantile fundus diseases will be explored in our future studies.

In summary, DeepQuality performed well in the multidimensional quality assessment of infantile fundus images in terms of illumination, clarity, and integrity. DeepQuality also provided comprehensive scoring for the overall quality of each image. More importantly, images with quality issues could be effectively enhanced by DeepQuality, which significantly improved ROP diagnosis. The integration of DeepQuality and AI diagnostic models can effectively improve the accuracy and robustness of models in real-world infant retinopathy screening. Given the pervasiveness of image quality issues across various medical specialties, this study may be an important reference for the development of other image-based AI disease screening systems.

## Methods

### Study design

**Fig. 3:**
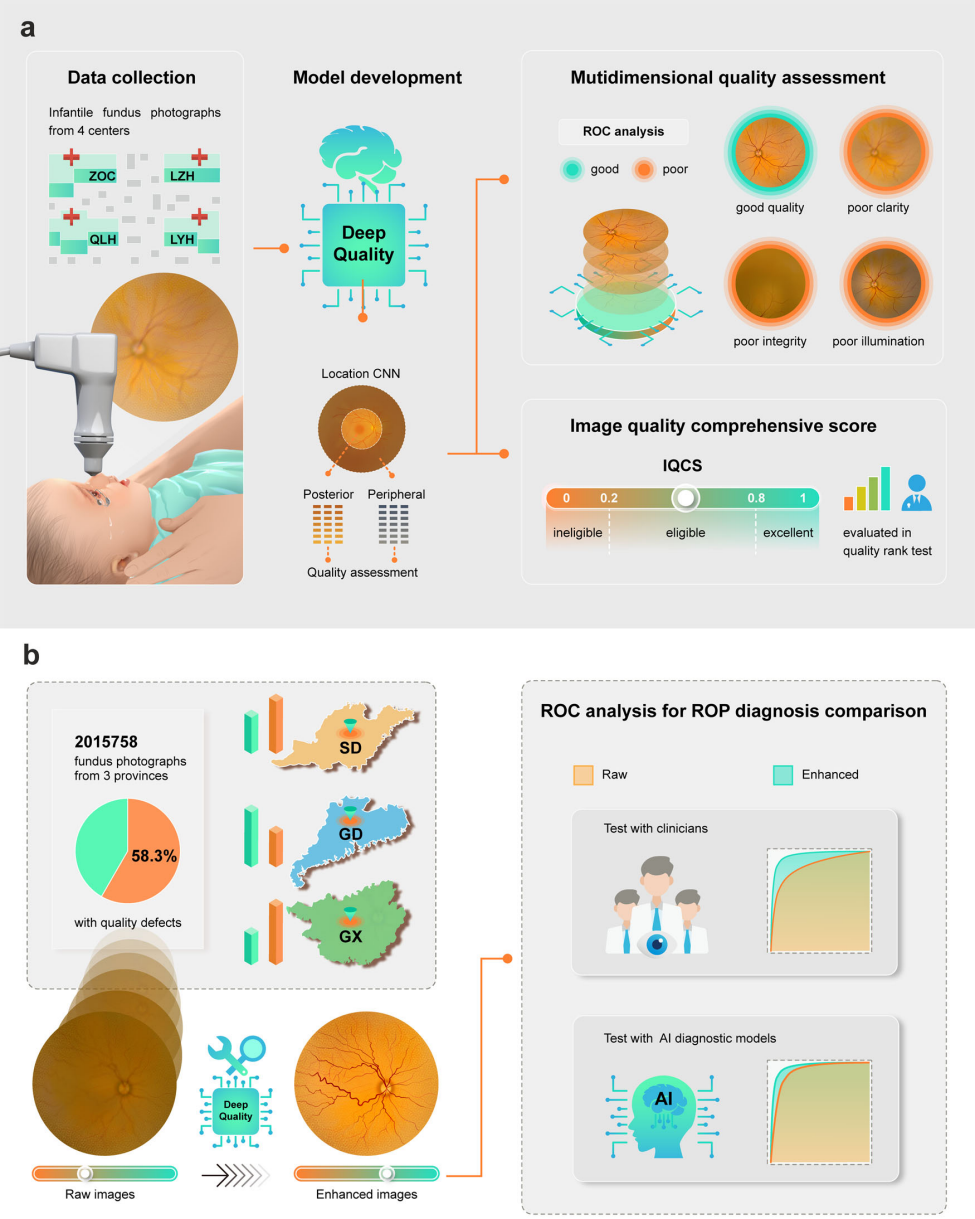
Overall workflow of DeepQuality development. **a** The development of quality assessment module. Infantile fundus images from four centers were collected and annotated to develop and evaluate quality assessment and scoring modules. **b** The real-world investigation and quality enhancement module. We investigated the image quality of infantile fundus photographs in real-world settings by DeepQuality. The quality enhancement module, which could enhance the quality based on the results of the quality assessment, was developed. The diagnostic performances of clinicians and AI diagnostic systems based on the raw and corresponding enhanced images were compared. IQCS image quality comprehensive score, SD Shandong Province, GX Guangxi Province, GD Guangdong Province, AI artificial intelligence.

The overall study design is shown in Fig. 3. We constructed a dataset with infantile fundus images acquired from the newborn fundus screening cohorts of Zhongshan Ophthalmic Center (ZOC), Maternal and Children’s Hospital of Liuzhou (LZH), Maternal and Children’s Hospital of Linyi (LYH), and Qilu Hospital (QLH) to develop and evaluate DeepQuality. These images were captured using Retcam (Natus Medical Inc., Pleasanton, USA) and PanoCam (Visunex Medical Systems Inc., San Ramon, USA). DeepQuality was designed to be a system consisting of the following three modules: quality assessment, quality scoring, and quality enhancement modules.

The study was approved by the Institutional Review Board of Zhongshan Ophthalmic Center at Sun Yat-sen University (IRB-ZOC-SYSU, ID: 2023KYP1029). All procedures were conducted in accordance with the tenets of the Declaration of Helsinki. Informed consent is waived because data are collected retrospectively and personal information is desensitized.

### Image quality annotation

Fundus images were first classified into posterior or peripheral images according to the integrity of the macula and optic disc. Then, all the images were labeled according to quality annotation criteria. Detailed definitions and typical examples of each quality factor are demonstrated in Supplementary Fig. 1. The proposed quality factors (integrity, illumination, and clarity) are well-established quality assessment aspects for fundus images in clinical practice^26^. For each quality factor, images were manually classified according to the criteria into two categories: “good” and “poor”. Two certified retinal experts with at least 5 years of clinical experience in pediatric ophthalmology were recruited to label all anonymized images independently. To ensure the reliability of image annotation, reference standards were determined only when both retinal experts reached consensus. All disputed images were submitted to another senior retinal expert with more than 10 years of clinical experience for arbitration.

### Development of the multidimensional quality assessment module of DeepQuality

Infantile fundus images collected from ZOC from January 2016 to December 2016 were randomly divided into the training set, development set, and internal test set at a ratio of 3:1:1. There was no patient overlap between these sets. All images were downsized to 299 × 299 pixels, and the pixel values were normalized to the interval [0, 1].

We used InceptionV3, a prominent deep convolutional neural network (CNN) architecture, to train our models. Corresponding to the annotation criteria (Supplementary Fig. 1), the image quality classification module of DeepQuality was composed of 1 model for differentiating the location (posterior and peripheral) of fundus images, 6 models for classifying different quality aspects of posterior fundus images, and 3 models for classifying different quality aspects of peripheral fundus images. Each model had one input and two outputs; the input of the model was a retinal image, and the outputs were a binary classification result and the corresponding probability of whether the quality of the input image was poor in the targeted aspect.

The Adam optimizer was used throughout the entire training procedure. The initial learning rate was set to 0.001, and the learning rate was decreased by a factor of 2 when the accuracy on the validation set stopped improving for 3 epochs to allow for fine learning. All the parameters were initialized with the default ImageNet weights. We trained the model for 50 epochs with a batch size of 64 and chose the model with optimal performance on the validation set. The development environment was based on Ubuntu 16.04.6 with an NVIDIA Tesla V100 PCle 32 GB. The versions of Python, TensorFlow, and Keras are 3.6.13, 1.13.1, and 2.3.1, respectively.

### External test of the quality assessment module

The performance of the quality assessment module of DeepQuality was externally tested using fundus images collected from LZH, LYH, and QLH from January 2022 to June 2022. The detailed information is summarized in Table 1.

### Visualization heatmap

The area of the image that the model focused on was highlighted using the Gradient-weighted Class Activation Map (Grad-CAM) visualization technique. Grad-CAM produces an activation heatmap based on a gradient. A greater value in the heatmap indicates a more important area for the model’s predictions. By adopting this technique, we can intuitively recognize the location of information used by the model to make decisions.

### Quality scoring module of DeepQuality

The overall quality of an image is determined by multiple factors, such as illumination, clarity, and integrity. To obtain a flexible evaluation metric for image quality, we defined the Image Quality Comprehensive Score (IQCS) to quantitatively reflect overall image quality. IQCS was calculated based on a linear weighting of the output probabilities of the quality assessment module. For posterior and peripheral fundus images, the IQCS is the ensemble of their respective quality aspects, and the detailed calculation of IQCS is presented in Eq. (1):

1 $${{\rm{IQCS}}}=1-\mathop{\sum }\limits_{i=1}^{k}{w}_{i}{p}_{i}$$

where *k* denotes the number of quality aspects. For posterior fundus images, *k* is 6 (macular clarity, macular illumination, optic-disc clarity, optic-disc illumination, remaining retina clarity, and remaining retina illumination), and for peripheral fundus images, *k* is 3 (illumination, clarity, and integrity). ${p}_{i}$ is the probability of predicting that an image is “poor” in terms of the *i*th quality aspect, and *w*_*i*_ indicates the weight of each aspect such that $\mathop{\sum }\nolimits_{i=1}^{k}{w}_{i}=1$.

In this study, the weight parameters were set such that *w*_1_ = *w*_2_ = …*w*_*n*_ and the IQCS ranged from 0 to 1, with a greater value indicating better overall quality and vice versa.

### Ranking test and threshold optimization for IQCS

To investigate the performance of the IQCS, two independent ranked test datasets, which consisted of 30 posterior and 30 peripheral fundus images, were constructed. For each ranked dataset, images were ranked in quality from best to worst by six retinal experts. A web-based interface, which presented experts with two images and prompted experts to “select the higher quality for the infant fundus screening”, was designed. After 15 rounds of pairwise comparisons, individual expert rankings of image quality were constructed with the ELO scoring scheme. The consensus ranking was based on the average ELO scores.

To investigate the effectiveness of the IQCS in ROP screening scenarios, we collected fundus images from routine ROP screening cohorts and constructed an ROP screening dataset. The gradability of fundus images was annotated according to new criteria. Detailed definitions of the annotation criteria are shown in Supplementary Table 5. Briefly, infantile fundus images were classified into excellent quality, eligible quality, and ineligible quality, in order of worsening quality defects and diminishing confidence of clinicians in their diagnosis of ROP. We attempted to find the optimal threshold for IQCS to distinguish the three quality categories via grid search. Briefly, the initial value of the lower threshold was 0.05 with a possible range of [0.05, 0.9]; the initial value of the upper threshold was 0.1, with a possible range of [0.1, 0.95]; and the upper and lower thresholds were then iterated to cover the entire range in steps of 0.05. The classification accuracy was calculated for all pairs of upper and lower thresholds, and the final pair with the highest accuracy was set as the IQCS threshold.

### A real-world investigation on the quality of infantile fundus images

Infantile fundus photographs from one ophthalmic hospital and two maternity and child healthcare hospitals were collected from January 2017 to December 2021. Then, we used DeepQuality to compare the proportions of poor illumination, poor clarity, and poor completeness of infant and child fundus images from different hospital sources. The distribution of IQCS among different hospitals was also investigated.

### Quality enhancement module of DeepQuality

For images with various quality defects, we developed a targeted quality enhancement module to improve the image quality. After the evaluation of the quality classification module and quantification modules, if the IQCS of the image is lower than the set threshold and the illumination of the image is classified as “poor”, we use the gamma transform to improve it (Eq. (2)). Moreover, to reduce the noise introduced by brightening the image and to preserve the edge information, the γ-transformed image is processed by a bilateral filter (Eq. (3)). If the clarity of the image is identified as “poor”, we use the CLAHE^27^ algorithm for the red and green channels to improve clarity:

2 $$Y={\left(\frac{I}{255}\right)}^{r}* 255$$

Gamma transform: I refers to the pixel value of the original image, *Y* is the value after the gamma transform, *r* is the transform coefficient, only the red and green channels are transformed, and the transform coefficients are 0.7 and 0.9.

3 $${I}^{p}=\frac{1}{{W}_{p}}\sum _{q\in S}{G}_{{\sigma }_{s}\left({||p}-{q||}\right)}{G}_{{\sigma }_{r}\left(|{I}_{p}-{I}_{q}|\right){I}_{p}}$$

Bilateral filter: the radius of the *S* domain is 10 pixels, the space sigma (*σ*_*s*_) in the Gaussian function is 30, and the range sigma (*σ*_*r*_) is 40.

### Comparisons for ROP diagnosis with or without the quality enhancement module

According to the International Committee for the Classification of ROP, the clinical features of ROP can be summarized as follows: (1) Basic lesions: an abnormal reaction to the end of immature vessels at the vascular-avascular juncture, with 5 stages. (2) Plus disease: abnormal dilatation and tortuosity of vessels in the posterior pole of the retina, including plus and pre-plus disease. In this study, images of stage 4 and stage 5 were not included. Two residents with 5 years of experience in ophthalmology annotated each fundus image by combining the results of binocular indirect ophthalmoscopy. If any of the abovementioned features were present in the image, it was labeled “ROP”, suggesting the need for further assessment. Conversely, an image without any visible features was labeled “Normal”. For each image, if the labels of the two residents agreed, the labels were accepted, and if they disagreed, another attending ophthalmologist with 10 years of clinical experience was consulted. The ROP screening database was constructed after this step.

After image annotation, 70 “ROP” images and 30 “Normal” images were randomly selected from the ROP screening dataset and formed an independent subset for clinician testing. Furthermore, images were processed by the quality enhancement module. Eight licensed ophthalmologists with similar clinical experience were recruited and randomly divided into two groups. One group performed the diagnosis task with raw images. Another group performed the diagnosis task with enhanced images. The accuracy, sensitivity, and specificity of the two groups were compared.

To investigate the assistance of quality enhancement on the model performance, we developed and evaluated ROP diagnostic models with raw images and corresponding enhanced images from ROP screening dataset (Supplementary Table 12), respectively. The performances were compared between different models. Two types of CNN architectures, InceptionV3 and DenseNet, were tested in this study. For both CNN architectures, the hyperparameters were fixed to explore the effectiveness of the quality enhancement module.

### Statistical analysis

The performance of the quality classification module in distinguishing poor-quality images in terms of each quality aspect was evaluated by sensitivity and specificity with 95% confidence intervals (CIs). Receiver operating characteristic (ROC) curves were plotted to show the performance of the quality classification module to assess image quality. Spearman’s rank correlation was used to evaluate the similarity between the IQCS and the consensus ranking of the images by the retinal experts. *p* < 0.05 (two-tailed) was considered to indicate statistical significance. Data were analyzed using Python 3.6.13.

### Reporting summary

Further information on research design is available in the Nature Research Reporting Summary linked to this article.

### Supplementary information

Supplementary information

Reporting Summary

## Data Availability

The medical records data reported in this study cannot be deposited in a public repository due to privacy concerns. Deidentified participant data will be made available upon reasonable request from the corresponding author (H.L., linht5@mail.sysu.edu.cn).

## Code availability

We made use of the open-source machine learning frameworks TensorFlow (https://github.com/tensorflow/tensorflow) to conduct the experiments. All the experiments and implementation details are available in the “Methods” section. The source code of the deep learning models is available upon reasonable request from the corresponding authors.
