# Supplementary material for: DeepQuality improves infant retinopathy screening
Source: NPJ Digit Med. 2023 Oct 16;6:192. doi: 10.1038/s41746-023-00943-3 (PMC10579317; doi:10.1038/s41746-023-00943-3)
Supplement: Supplementary file 1 — Supplementary information [file 41746_2023_943_MOESM1_ESM.pdf]

Supplementary information

DeepQuality Improves Infant Retinopathy Screening

Contents

Supplementary Figure 1. 3

Supplementary Figure 2. 5

Supplementary Figure 3. 6

Supplementary Figure 4. 7

Supplementary Figure 5. 8

Supplementary Table 1. 9

Supplementary Table 2. 10

Supplementary Table 3. 11

Supplementary Table 4. 12

Supplementary Table 5. 13

Supplementary Table 6. 14

Supplementary Table 7. 14

Supplementary Table 8. 15

Supplementary Table 9. 19

Supplementary Table 10. 20

Supplementary Table 11. 21

Supplementary Table 12. 22

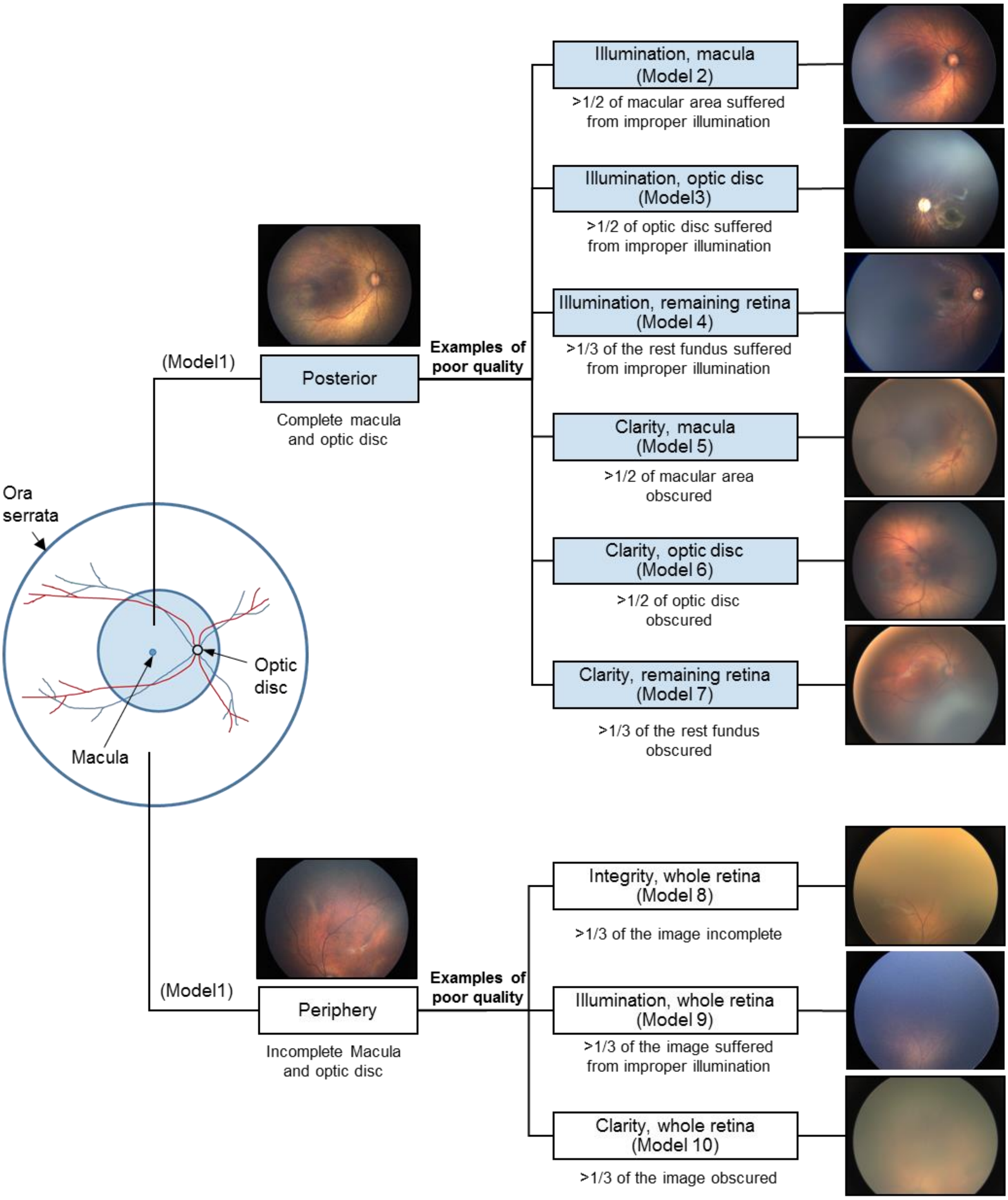

Supplementary Figure 1. Definitions and examples of different infantile fundus image quality groups. Fundus images were first classified into posterior and peripheral images based on the integrity of the macula and optic disc. For posterior images, quality was annotated in terms of both clinical factors (illumination and clarity) and structural factors (macula, optic disc, and remaining retina). For peripheral images, quality was annotated only in terms of clinical factors (integrity, illumination, and clarity) because of the lack of anatomical landmarks. The macula area was defined as the area within a

1.5-disc diameter of the fovea. Improper illumination was defined as illumination that was either too bright or too dark to accurately identify retinal structures or abnormalities.

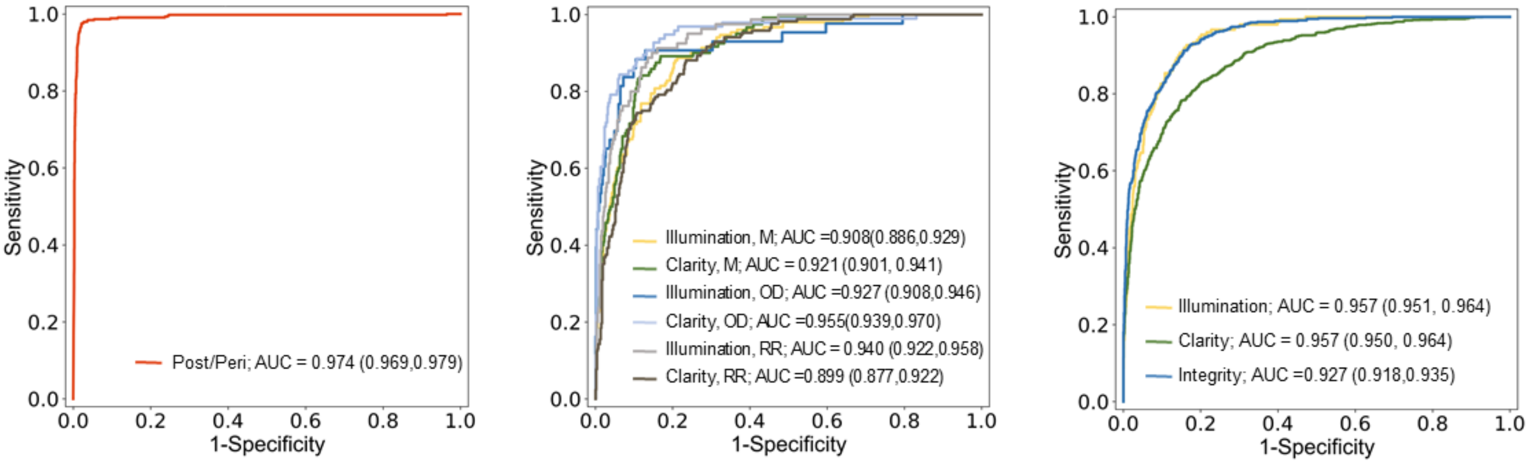

Supplementary Figure 2. Performance of the quality assessment module in internal tests. In the internal test dataset, the assessment module achieved an AUC of 0.974 for distinguishing the location of fundus images (A). For posterior images, the assessment module achieved AUCs of 0.899-0.955 for detecting various quality defects (B). For peripheral images, the assessment module achieved AUCs of 0.927-0.957 for detecting various quality defects (C). Post, posterior; Peri, peripheral; M, macular; OD, optic disk; RR, remaining retina.

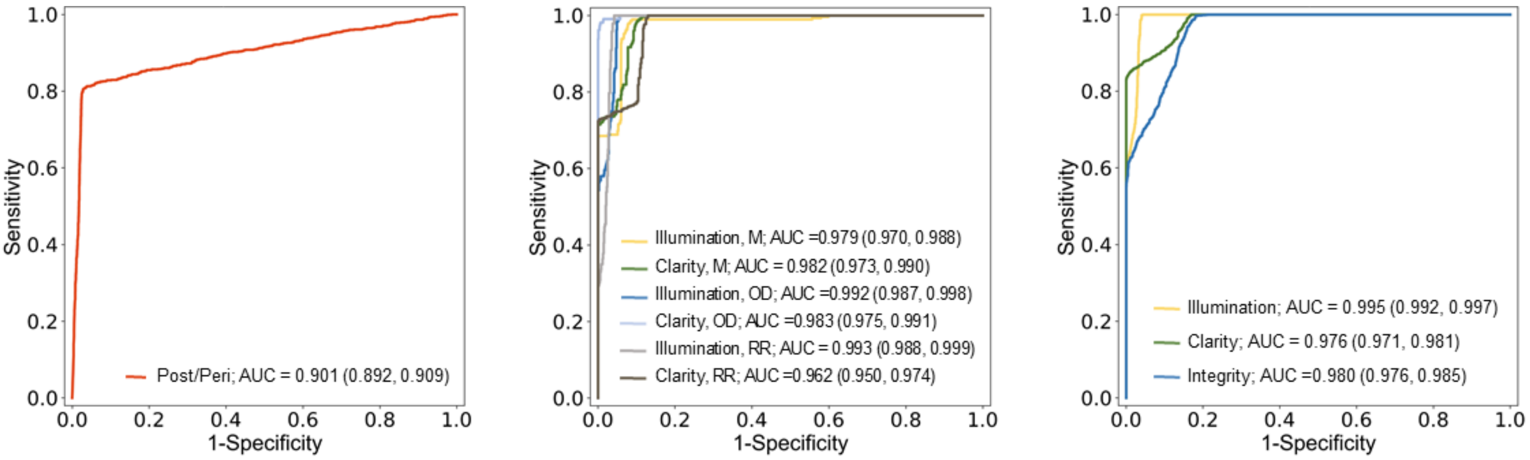

Supplementary Figure 3. Performance of the quality assessment module in the external test (LZH). In the external test dataset from ZOC, the assessment module achieved an AUC of 0.901 for distinguishing the location of fundus images (A). For posterior images, the assessment module achieved AUCs of 0.962-0.993 for detecting various quality defects (B). For peripheral images, the assessment module achieved AUCs of 0.976-0.995 for detecting various quality defects (C). Post, posterior; Peri, peripheral; M, macular; OD, optic disk; RR, remaining retina; LZH, Maternal and Children's Hospital of Liuzhou.

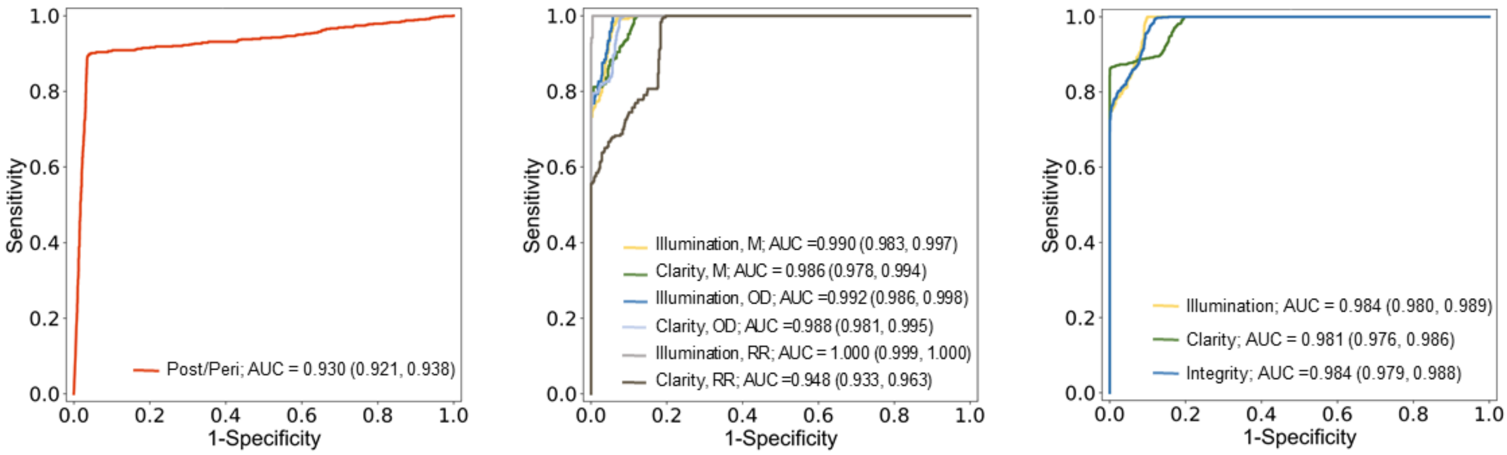

Supplementary Figure 4. Performance of the quality assessment module in the external test (LYH). In the external test dataset from LYH, the assessment module achieved an AUC of 0.930 for distinguishing the location of fundus images (A). For posterior images, the assessment module achieved AUCs of 0.948-1.000 for detecting various quality defects (B). For peripheral images, the assessment module achieved AUCs of 0.981-0.984 for detecting various quality defects (C). Post, posterior; Peri, peripheral; M, macular; OD, optic disk; RR, remaining retina; LYH, Maternal and Children's Hospital of Linyi.

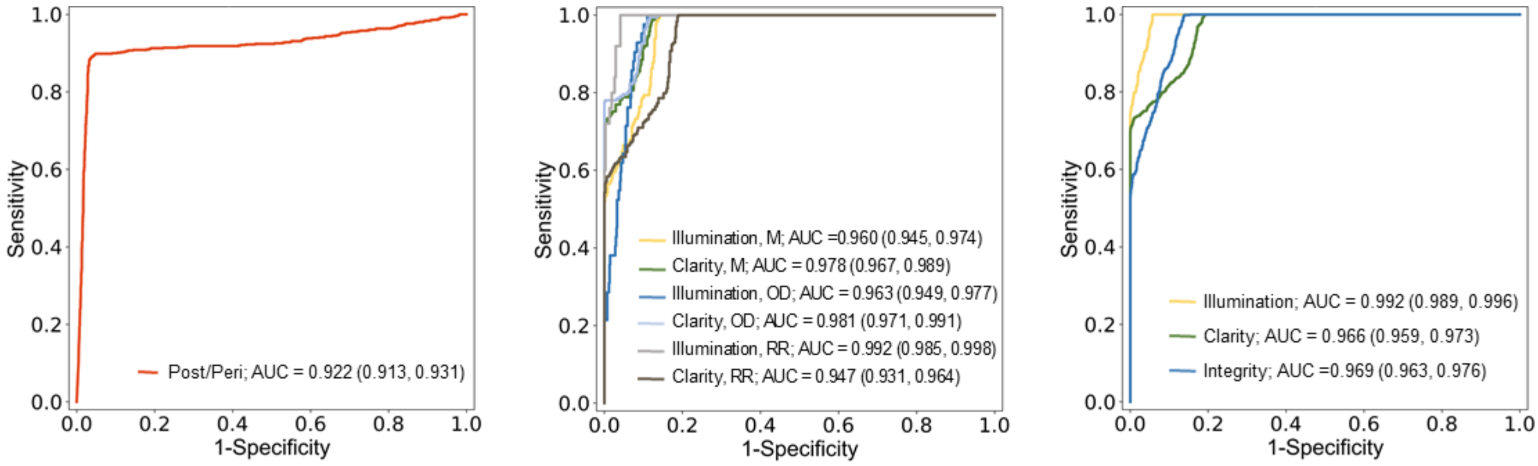

Supplementary Figure 5. Performance of the quality assessment module in the external test (QLH). In the external test dataset from LYH, the assessment module achieved an AUC of 0.922 for distinguishing the location of fundus images (A). For posterior images, the assessment module achieved AUCs of 0.947-0.992 for detecting various quality defects (B). For peripheral images, the assessment module achieved AUCs of 0.966-0.992 for detecting various quality defects (C). Post, posterior; Peri, peripheral; M, macular; OD, optic disk; RR, remaining retina; QLH, Qilu Hospital.

Supplementary Table 1. Demographic characteristics of the datasets.

	Development dataset (N = 15998)	Internal test dataset (N = 4001)	External test dataset (N = 12753)		
			LZH	LYH	QLH
Subjects (Male)	2018 (1088)	496 (256)	599 (316)	492 (268)	425 (235)
Gestational age (w)	37.9±2.3	38.0±2.4	37.9±2.5	39.0±1.5	38.8±1.6
Birth weight (g)	3034.8±596.3	3026.4±558.6	3017.6±618.6	3456.4±498.2	3445.2±518.5
Ethnicity					
Han	1998	481	299	485	420
Zhuang	-	-	111	-	-
Miao	-	-	87	-	-
Yao	-	-	71	-	-
Other	20	15	31	7	5

Notes: N, number; w, week; g, gram; LZH, Maternal and Children’s Hospital of Liuzhou; LYH, Maternal and Children’s Hospital of Linyi; QLH, Qilu Hospital.

Supplementary Table 2. Model performance in the internal test.

Model		Sensitivity (95% CI)	Specificity (95% CI)	Accuracy (95% CI)	AUC (95% CI)
Location		0.882 (0.872, 0.892)	0.960 (0.954, 0.966)	0.946 (0.939, 0.953)	0.974 (0.969, 0.979)
Posterior	Illumination, macula	0.788 (0.758, 0.818)	0.856 (0.830, 0.882)	0.841 (0.814, 0.868)	0.908 (0.886, 0.929)
	Illumination, optic disc	0.837 (0.810, 0.865)	0.906 (0.884, 0.928)	0.902 (0.880, 0.924)	0.927 (0.908, 0.946)
	Illumination, remaining retina	0.800 (0.770, 0.830)	0.889 (0.866, 0.912)	0.879 (0.855, 0.903)	0.940 (0.922, 0.958)
	Clarity, macula	0.883 (0.859, 0.907)	0.833 (0.805, 0.861)	0.842 (0.815, 0.869)	0.921 (0.901, 0.941)
	Clarity, optic disc	0.823 (0.795, 0.851)	0.941 (0.924, 0.959)	0.925 (0.905, 0.945)	0.955 (0.939, 0.970)
	Clarity, remaining retina	0.804 (0.774, 0.834)	0.808 (0.779, 0.837)	0.807 (0.778, 0.836)	0.899 (0.877, 0.922)
Peripheral	Integrity	0.860 (0.848, 0.872)	0.826 (0.813, 0.839)	0.833 (0.820, 0.846)	0.927 (0.918, 0.935)
	Illumination	0.913 (0.903, 0.923)	0.884 (0.873, 0.895)	0.887 (0.876, 0.898)	0.957 (0.951, 0.964)
	Clarity	0.862 (0.850, 0.879)	0.898 (0.888, 0.908)	0.884 (0.873, 0.895)	0.957 (0.950, 0.964)

Notes: CI, confidence interval; Location, posterior or peripheral; AUC, area under the receiver operating characteristic curve.

Supplementary Table 3. Model performance in the external test (LZH).

Model		Sensitivity (95% CI)	Specificity (95% CI)	Accuracy (95% CI)	AUC (95% CI)
Location		0.882 (0.873, 0.891)	0.983 (0.979, 0.987)	0.961 (0.956, 0.967)	0.901 (0.892, 0.909)
Posterior	Illumination, macula	0.852 (0.830, 0.874)	0.924 (0.907, 0.941)	0.908 (0.890, 0.926)	0.979 (0.970, 0.988)
	Illumination, optic disc	0.886 (0.866, 0.906)	0.958 (0.945, 0.971)	0.95 (0.936, 0.964)	0.992 (0.987, 0.998)
	Illumination, remaining retina	0.837 (0.814, 0.860)	0.976 (0.966, 0.986)	0.969 (0.958, 0.980)	0.993 (0.988, 0.999)
	Clarity, macula	0.864 (0.843, 0.886)	0.905 (0.887, 0.923)	0.895 (0.876, 0.914)	0.982 (0.973, 0.990)
	Clarity, optic disc	0.833 (0.810, 0.856)	0.929 (0.913, 0.945)	0.906 (0.888, 0.924)	0.983 (0.975, 0.991)
	Clarity, remaining retina	0.837 (0.814, 0.860)	0.865 (0.844, 0.886)	0.856 (0.834, 0.878)	0.962 (0.950, 0.974)
Peripheral	Integrity	0.879 (0.869, 0.889)	0.924 (0.916, 0.932)	0.911 (0.902, 0.920)	0.980 (0.976, 0.985)
	Illumination	0.928 (0.920, 0.936)	0.959 (0.953, 0.965)	0.954 (0.947, 0.961)	0.995 (0.992, 0.997)
	Clarity	0.879 (0.869, 0.889)	0.871 (0.860, 0.882)	0.875 (0.865, 0.885)	0.976 (0.971, 0.981)

Notes: CI, confidence interval; Location, posterior or peripheral; AUC, area under the receiver operating characteristic curve. LZH, Maternal and Children's Hospital of Liuzhou.

Supplementary Table 4. Model performance in the external test (LYH).

Model		Sensitivity (95% CI)	Specificity (95% CI)	Accuracy (95% CI)	AUC (95% CI)
Location		0.891 (0.881, 0.901)	0.964 (0.958, 0.970)	0.949 (0.942, 0.956)	0.930 (0.921, 0.938)
Posterior	Illumination, macula	0.814 (0.788, 0.840)	0.969 (0.957, 0.981)	0.923 (0.905, 0.941)	0.990 (0.983, 0.997)
	Illumination, optic disc	0.851 (0.827, 0.875)	0.970 (0.958, 0.982)	0.953 (0.939, 0.967)	0.992 (0.986, 0.998)
	Illumination, remaining retina	0.884 (0.862, 0.906)	1.000 (1.000, 1.000)	0.990 (0.983, 0.997)	1.000 (0.999, 1.000)
	Clarity, macula	0.896 (0.875, 0.917)	0.933 (0.916, 0.950)	0.922 (0.904, 0.940)	0.986 (0.978, 0.994)
	Clarity, optic disc	0.848 (0.824, 0.872)	0.945 (0.930, 0.961)	0.918 (0.899, 0.937)	0.988 (0.981, 0.995)
	Clarity, remaining retina	0.823 (0.797, 0.849)	0.837 (0.809, 0.861)	0.833 (0.807, 0.859)	0.948 (0.933, 0.963)
Peripheral	Integrity	0.904 (0.894, 0.914)	0.916 (0.906, 0.926)	0.913 (0.903, 0.923)	0.984 (0.979, 0.988)
	Illumination	0.932, (0.923, 0.941)	0.915, (0.905, 0.925)	0.917, (0.907, 0.927)	0.984 (0.980, 0.989)
	Clarity	0.895, (0.884, 0.906)	0.876, (0.864, 0.888)	0.885, (0.874, 0.896)	0.981 (0.976, 0.986)

Notes: CI, confidence interval; Location, posterior or peripheral; AUC, area under the receiver operating characteristic curve; LYH, Maternal and Children's Hospital of Linyi.

Supplementary Table 5. Model performance in the external test (QLH).

Model		Sensitivity (95% CI)	Specificity (95% CI)	Accuracy (95% CI)	AUC (95% CI)
Location		0.883 (0.872, 0.894)	0.968 (0.962, 0.974)	0.950 (0.943, 0.957)	0.922 (0.913, 0.931)
Posterior	Illumination, macula	0.794 (0.764, 0.824)	0.885 (0.862, 0.908)	0.868 (0.843, 0.893)	0.960 (0.945, 0.974)
	Illumination, optic disc	0.857 (0.831, 0.883)	0.927 (0.908, 0.946)	0.923 (0.903, 0.943)	0.963 (0.949, 0.977)
	Illumination, remaining retina	0.800 (0.771, 0.829)	0.977 (0.966, 0.988)	0.971 (0.959, 0.983)	0.992 (0.985, 0.998)
	Clarity, macula	0.885 (0.862, 0.908)	0.905 (0.884, 0.927)	0.900 (0.878, 0.922)	0.978 (0.967, 0.989)
	Clarity, optic disc	0.822 (0.794, 0.850)	0.929 (0.910, 0.948)	0.900 (0.878, 0.922)	0.981 (0.971, 0.991)
	Clarity, remaining retina	0.798 (0.769, 0.828)	0.844 (0.817, 0.871)	0.829 (0.801, 0.857)	0.947 (0.931, 0.964)
Peripheral	Integrity	0.850 (0.837, 0.864)	0.914 (0.903, 0.925)	0.896 (0.884, 0.908)	0.969 (0.963, 0.976)
	Illumination	0.904 (0.893, 0.915)	0.963 (0.956, 0.970)	0.954 (0.946, 0.962)	0.992 (0.989, 0.996)
	Clarity	0.860 (0.847, 0.873)	0.857 (0.844, 0.870)	0.859 (0.846, 0.872)	0.966 (0.959, 0.973)

Notes: CI, confidence interval; Location, posterior or peripheral; AUC, area under the receiver operating characteristic curve; QLH, Qilu Hospital.

Supplementary Table 6. Grading principles for infantile fundus images during IQCS threshold exploration.

Quality classification	Quality defects	Description
Excellent	No noticeable defects	Image quality sufficient to diagnose ROP with confidence
Eligible	Noticeable defects	Image quality sufficient to diagnose ROP, but with limited confidence
Ineligible	Severe defects	Image quality is unacceptable to diagnose ROP

Supplementary Table 7. Number of images in the ROP screening database for each quality classification.

Quality classification	Posterior	Peripheral	Total
Excellent	589	573	1162
Eligible	446	5776	6223
Ineligible	110	905	1015

Note: ROP, retinopathy of prematurity.

Supplementary Table 8. Grid search for the optimal threshold to distinguish quality grading.

Upper bound of threshold	Lower bound of threshold	Accuracy for posterior images	Accuracy for peripheral images
0.05	0.10	0.484	0.469
0.05	0.15	0.490	0.475
0.05	0.20	0.499	0.487
0.05	0.25	0.511	0.498
0.05	0.30	0.521	0.510
0.05	0.35	0.537	0.559
0.05	0.40	0.550	0.615
0.05	0.45	0.567	0.668
0.05	0.50	0.585	0.726
0.05	0.55	0.613	0.779
0.05	0.60	0.623	0.820
0.05	0.65	0.645	0.852
0.05	0.70	0.696	0.856
0.05	0.75	0.732	0.851
0.05	0.80	0.752	0.850
0.05	0.85	0.726	0.832
0.05	0.90	0.718	0.806
0.05	0.95	0.696	0.763
0.10	0.15	0.520	0.474
0.10	0.20	0.529	0.487
0.10	0.25	0.542	0.498
0.10	0.30	0.552	0.509
0.10	0.35	0.567	0.559
0.10	0.40	0.580	0.614
0.10	0.45	0.597	0.667
0.10	0.50	0.615	0.726
0.10	0.55	0.643	0.778
0.10	0.60	0.653	0.819
0.10	0.65	0.675	0.852
0.10	0.70	0.726	0.856
0.10	0.75	0.762	0.851
0.10	0.80	0.782	0.850
0.10	0.85	0.756	0.832
0.10	0.90	0.748	0.805
0.10	0.95	0.726	0.762
0.15	0.20	0.547	0.481
0.15	0.25	0.560	0.493
0.15	0.30	0.570	0.504
0.15	0.35	0.586	0.553

0.15	0.40	0.599	0.609
0.15	0.45	0.616	0.662
0.15	0.50	0.633	0.720
0.15	0.55	0.662	0.773
0.15	0.60	0.672	0.814
0.15	0.65	0.693	0.856
0.15	0.70	0.745	0.850
0.15	0.75	0.781	0.856
0.15	0.80	0.801	0.844
0.15	0.85	0.775	0.827
0.15	0.90	0.766	0.800
0.15	0.95	0.745	0.757
0.20	0.25	0.599	0.480
0.20	0.30	0.609	0.492
0.20	0.35	0.625	0.541
0.20	0.40	0.638	0.597
0.20	0.45	0.655	0.650
0.20	0.50	0.672	0.708
0.20	0.55	0.701	0.761
0.20	0.60	0.711	0.802
0.20	0.65	0.732	0.844
0.20	0.70	0.784	0.856
0.20	0.75	0.819	0.843
0.20	0.80	**0.842**	**0.858**
0.20	0.85	0.814	0.814
0.20	0.90	0.805	0.788
0.20	0.95	0.784	0.745
0.25	0.30	0.610	0.480
0.25	0.35	0.626	0.529
0.25	0.40	0.639	0.585
0.25	0.45	0.656	0.637
0.25	0.50	0.673	0.696
0.25	0.55	0.702	0.749
0.25	0.60	0.712	0.789
0.25	0.65	0.734	0.832
0.25	0.70	0.785	0.846
0.25	0.75	0.821	0.831
0.25	0.80	0.841	0.820
0.25	0.85	0.815	0.802
0.25	0.90	0.807	0.775
0.25	0.95	0.785	0.733
0.30	0.35	0.628	0.517
0.30	0.40	0.640	0.572
0.30	0.45	0.658	0.625

0.30	0.50	0.675	0.684
0.30	0.55	0.703	0.736
0.30	0.60	0.713	0.777
0.30	0.65	0.735	0.820
0.30	0.70	0.787	0.834
0.30	0.75	0.822	0.819
0.30	0.80	0.840	0.808
0.30	0.85	0.817	0.790
0.30	0.90	0.808	0.763
0.30	0.95	0.787	0.720
0.35	0.40	0.639	0.522
0.35	0.45	0.656	0.575
0.35	0.50	0.673	0.633
0.35	0.55	0.702	0.686
0.35	0.60	0.712	0.727
0.35	0.65	0.734	0.769
0.35	0.70	0.785	0.783
0.35	0.75	0.821	0.768
0.35	0.80	0.841	0.757
0.35	0.85	0.815	0.739
0.35	0.90	0.807	0.713
0.35	0.95	0.785	0.670
0.40	0.45	0.650	0.517
0.40	0.50	0.668	0.576
0.40	0.55	0.696	0.629
0.40	0.60	0.706	0.669
0.40	0.65	0.728	0.712
0.40	0.70	0.779	0.726
0.40	0.75	0.815	0.711
0.40	0.80	0.835	0.700
0.40	0.85	0.809	0.682
0.40	0.90	0.801	0.655
0.40	0.95	0.779	0.613
0.45	0.50	0.653	0.518
0.45	0.55	0.682	0.571
0.45	0.60	0.692	0.611
0.45	0.65	0.713	0.654
0.45	0.70	0.765	0.668
0.45	0.75	0.801	0.653
0.45	0.80	0.821	0.642
0.45	0.85	0.795	0.624
0.45	0.90	0.787	0.597
0.45	0.95	0.765	0.554
0.50	0.55	0.670	0.506

0.50	0.60	0.681	0.546
0.50	0.65	0.702	0.589
0.50	0.70	0.754	0.603
0.50	0.75	0.789	0.588
0.50	0.80	0.809	0.577
0.50	0.85	0.784	0.559
0.50	0.90	0.775	0.532
0.50	0.95	0.754	0.489
0.55	0.60	0.649	0.485
0.55	0.65	0.670	0.528
0.55	0.70	0.722	0.542
0.55	0.75	0.758	0.527
0.55	0.80	0.778	0.516
0.55	0.85	0.752	0.498
0.55	0.90	0.744	0.471
0.55	0.95	0.722	0.428
0.60	0.65	0.650	0.476
0.60	0.70	0.702	0.490
0.60	0.75	0.738	0.475
0.60	0.80	0.758	0.464
0.60	0.85	0.732	0.446
0.60	0.90	0.723	0.420
0.60	0.95	0.702	0.377
0.65	0.70	0.676	0.435
0.65	0.75	0.712	0.420
0.65	0.80	0.732	0.409
0.65	0.85	0.706	0.391
0.65	0.90	0.698	0.365
0.65	0.95	0.676	0.322
0.70	0.75	0.649	0.390
0.70	0.80	0.669	0.379
0.70	0.85	0.643	0.361
0.70	0.90	0.635	0.334
0.70	0.95	0.613	0.292
0.75	0.80	0.629	0.374
0.75	0.85	0.603	0.356
0.75	0.90	0.595	0.329
0.75	0.95	0.573	0.287
0.80	0.85	0.577	0.350
0.80	0.90	0.569	0.323
0.80	0.95	0.547	0.280
0.85	0.90	0.547	0.318
0.85	0.95	0.526	0.275
0.90	0.95	0.526	0.269

Supplementary Table 9. The proportion of quality defects of infantile fundus images from different hospitals.

		OH (N =244270)		MCH 1 (N = 600813)		MCH 2 (N =1170665)	
		AQ group	PQ group	AQ group	PQ group	AQ group	PQ group
Posterior	Illumination, macula	36979 (73.5%)	13332 (26.5%)	81486 (53.5%)	70825 (46.5%)	228318 (56.2%)	178155 (43.8%)
	Illumination, optic disc	43418 (86.3%)	6893 (13.7%)	131515 (68.6%)	20796 (31.4%)	259028 (63.7%)	147445 (36.3%)
	Illumination, remaining retina	46538 (92.5%)	3773 (7.5%)	128543 (84.4%)	23768 (15.6%)	252826 (62.2%)	153647 (37.8%)
	Clarity, macula	38799 (77.1%)	11512 (22.9%)	111351 (73.1%)	40960 (26.9%)	244558 (60.2%)	161915 (39.8%)
	Clarity, optic disc	43657 (86.8%)	6654 (13.2%)	128082 (84.1%)	24229 (15.9%)	218454 (53.7%)	188019 (46.3%)
	Clarity, remaining retina	34010 (67.6%)	16301 (32.4%)	96108 (63.1%)	56203 (36.9%)	146790 (63.9%)	259683 (36.1%)
Peripheral	Integrity, whole retina	152840 (78.8%)	41119 (21.2%)	204068 (45.5%)	244434 (54.5%)	499473 (65.4%)	264719 (34.6%)
	Illumination, whole retina	162344 (83.7%)	31615 (16.3%)	257440 (57.4%)	191062 (42.6%)	610402 (79.9%)	153790 (20.1%)
	Clarity, whole retina	140038 (72.2%)	53921 (27.8%)	313503 (69.9%)	134999 (30.1%)	473291 (61.9%)	290901 (38.1%)

Notes: OH, Ophthalmic Hospital; MCH, Maternity and Children Hospital; N, number.

Supplementary Table 10. The IQCS distribution of infantile fundus images from different hospitals.

	IQCS	OH (N =244,270)	MCH 1 (N =600,813)	MCH 2 (N =1,170,665)	P
Posterior	≥0.8	30388 (60.4%)	60163 (39.5%)	123267 (30.3%)	<0.01
	0.2-0.8	17860 (35.5%)	82400 (54.1%)	247098 (60.8%)	<0.01
	≤0.2	2064 (4.1%)	9748 (6.4%)	36108 (8.9%)	<0.01
Peripheral	≥0.8	120642 (62.2%)	146212 (32.6%)	359003 (47.0%)	<0.01
	0.2-0.8	51205 (26.4%)	218869 (48.8%)	282370 (37.0%)	<0.01
	≤0.2	22111 (11.4%)	83421 (18.6%)	122819 (16.0%)	<0.01

Notes: OH, Ophthalmic Hospital; MCH, Maternity and Children Hospital; N, number; P, indicated the pairwise P value among OH, MCH 1 and MCH2 calculated by the two-proportion z test.

Supplementary Table 11. Model performance in randomized inspection of large-scale real-world dataset.

	Model	Sensitivity (95% CI)	Specificity (95% CI)	Accuracy (95% CI)	AUC (95% CI)
Posterior	Illumination, macula	0.929 (0.896, 0.961)	0.936 (0.911, 0.962)	0.933 (0.913, 0.953)	0.947 (0.927, 0.968)
	Illumination, optic disc	0.955 (0.923, 0.988)	0.912 (0.886, 0.938)	0.923 (0.902, 0.945)	0.947 (0.922, 0.972)
	Illumination, remaining retina	0.778 (0.687, 0.868)	0.990 (0.981, 0.998)	0.962 (0.946, 0.977)	0.926 (0.886, 0.966)
	Clarity, macula	0.815 (0.766, 0.865)	0.886 (0.853, 0.918)	0.858 (0.830, 0.886)	0.885 (0.855, 0.915)
	Clarity, optic disc	0.881 (0.831, 0.931)	0.784 (0.746, 0.823)	0.810 (0.779, 0.841)	0.915 (0.884, 0.946)
	Clarity, remaining retina	0.887 (0.852, 0.923)	0.824 (0.780, 0.867)	0.857 (0.829, 0.885)	0.871 (0.842, 0.900)
Peripheral	Integrity	0.821 (0.786, 0.856)	0.896 (0.877, 0.916)	0.871 (0.854, 0.889)	0.869 (0.846, 0.891)
	Illumination	0.803 (0.759, 0.847)	0.935 (0.920, 0.950)	0.906 (0.890, 0.921)	0.857 (0.830, 0.885)
	Clarity	0.822 (0.789, 0.855)	0.922 (0.905, 0.940)	0.885 (0.868, 0.902)	0.890 (0.871, 0.910)

Notes: CI, confidence interval; Location, posterior or peripheral; AUC, area under the receiver operating characteristic curve.

Supplementary Table 12. Numbers of retinal images used in ROP classification.

	Test for clinicians	Test for AI diagnostic models		
		Development dataset	Internal test dataset	External test dataset
ROP	70	1003	400	798
Normal	30	2234	1475	1475
Total	100	3237	1875	2273

Notes: ROP, retinopathy of prematurity; AI, artificial intelligence.
