# Supplementary material for: DeepQuality improves infant retinopathy screening
Source: NPJ Digit Med. 2023 Oct 16;6:192. doi: 10.1038/s41746-023-00943-3 (PMC10579317; doi:10.1038/s41746-023-00943-3)
Supplement: Supplementary file 2 — Reporting Summary [file 41746_2023_943_MOESM2_ESM.pdf]

nature portfolio

Corresponding author(s): Haotian Lin

Last updated by author(s): Aug 17, 2023

Reporting Summary

Nature Portfolio wishes to improve the reproducibility of the work that we publish. This form provides structure for consistency and transparency in reporting. For further information on Nature Portfolio policies, see our Editorial Policies and the Editorial Policy Checklist.

Statistics

For all statistical analyses, confirm that the following items are present in the figure legend, table legend, main text, or Methods section.

n/a	Confirmed	
☐	☒	The exact sample size (*n*) for each experimental group/condition, given as a discrete number and unit of measurement
☐	☒	A statement on whether measurements were taken from distinct samples or whether the same sample was measured repeatedly
☐	☒	The statistical test(s) used AND whether they are one- or two-sided<br>*Only common tests should be described solely by name; describe more complex techniques in the Methods section.*
☒	☐	A description of all covariates tested
☐	☒	A description of any assumptions or corrections, such as tests of normality and adjustment for multiple comparisons
☐	☒	A full description of the statistical parameters including central tendency (e.g. means) or other basic estimates (e.g. regression coefficient) AND variation (e.g. standard deviation) or associated estimates of uncertainty (e.g. confidence intervals)
☐	☒	For null hypothesis testing, the test statistic (e.g. *F*, *t*, *r*) with confidence intervals, effect sizes, degrees of freedom and *P* value noted<br>*Give P values as exact values whenever suitable.*
☒	☐	For Bayesian analysis, information on the choice of priors and Markov chain Monte Carlo settings
☐	☒	For hierarchical and complex designs, identification of the appropriate level for tests and full reporting of outcomes
☐	☒	Estimates of effect sizes (e.g. Cohen's *d*, Pearson's *r*), indicating how they were calculated

Our web collection on statistics for biologists contains articles on many of the points above.

Software and code

Policy information about availability of computer code

Data collection	No software was used.
Data analysis	The performance of the quality classification module in distinguishing poor-quality images in terms of each quality aspect was evaluated by sensitivity and specificity with 95% confidence intervals (CIs). Receiver operating characteristic (ROC) curves were plotted to show the performance of the quality classification module to assess image quality. Spearman's rank correlation was used to evaluate the similarity between the IQCS and the consensus ranking of the images by the retinal experts. P < 0.05 (two-tailed) was considered to indicate statistical significance. Data were analyzed using Python 3.6.13.

For manuscripts utilizing custom algorithms or software that are central to the research but not yet described in published literature, software must be made available to editors and reviewers. We strongly encourage code deposition in a community repository (e.g. GitHub). See the Nature Portfolio guidelines for submitting code & software for further information.

Data

Policy information about availability of data

All manuscripts must include a data availability statement. This statement should provide the following information, where applicable:

- Accession codes, unique identifiers, or web links for publicly available datasets
- A description of any restrictions on data availability
- For clinical datasets or third party data, please ensure that the statement adheres to our policy

The medical records data reported in this study cannot be deposited in a public repository due to privacy concerns. Deidentified participant data will be made available upon reasonable request from the corresponding author (HTL, linht5@mail.sysu.edu.cn).

Research involving human participants, their data, or biological material

Policy information about studies with human participants or human data. See also policy information about sex, gender (identity/presentation), and sexual orientation and race, ethnicity and racism.

Reporting on sex and gender	We identified the sexes of infants by their sexual characteristics at birth. We did not perform sex-based analysis. During the development and validation of DeepQulaity, 32112 fundus images from 4030 infants (2163 males, 1867 females) were used.
Reporting on race, ethnicity, or other socially relevant groupings	The proportion of ethnic minorities in the external test dataset of LZH reaches 50.1%, including Zhuang, Miao and Yao, etc. This part of data was used to verify the generalization ability of DeepQuality.
Population characteristics	The basic characteristics of participants was summarized in Table S1. Data on gestational age and birth weight were collected from EMRs.
Recruitment	The infantile fundus photographs of participants were retrospectively collected from January 2016 to June 2022,in Zhongshan Ophthalmic Center (ZOC), Maternal and Children's Hospital of Liuzhou (LZH), Maternal and Children's Hospital of Linyi (LYH), and Qilu Hospital (QLH).
Ethics oversight	The study was approved by the Institutional Review Board of Zhongshan Ophthalmic Center at Sun Yat-sen University . All procedures were conducted in accordance with the tenets of the Declaration of Helsinki.

Note that full information on the approval of the study protocol must also be provided in the manuscript.

Field-specific reporting

Please select the one below that is the best fit for your research. If you are not sure, read the appropriate sections before making your selection.

☒ Life sciences ☐ Behavioural & social sciences ☐ Ecological, evolutionary & environmental sciences

For a reference copy of the document with all sections, see nature.com/documents/nr-reporting-summary-flat.pdf

Life sciences study design

All studies must disclose on these points even when the disclosure is negative.

Sample size	A total of 2056260 infantile fundus images were enrolled for DeepQuality development. Among them, 32112 fundus images were labeled according to quality annotation criteria and used to develop and evaluate the quality assessment module; 2015748 fundus images were enrolled to investigate the real-world image quality distribution; and 8400 fundus images were labeled according to quality grading and characteristics of ROP, then used to develop the quality scoring module and perform the ROP diagnostic test after quality enhancement.
Data exclusions	To simulate the real-world setting situation, there is no data excluded in this study.
Replication	We used InceptionV3, a prominent deep convolutional neural network (CNN) architecture, to train our models. Corresponding to the annotation criteria (Figure S1), the image quality classification module of DeepQuality was composed of 1 model for differentiating the location (posterior and peripheral) of fundus images, 6 models for classifying different quality aspects of posterior fundus images, and 3 models for classifying different quality aspects of peripheral fundus images. Each model had one input and two outputs; the input of the model was a retinal image, and the outputs were a binary classification result and the corresponding probability of whether the quality of the input image was poor in the targeted aspect.<br>The Adam optimizer was used throughout the entire training procedure. The initial learning rate was set to 0.001, and the learning rate was decreased by a factor of 2 when the accuracy on the validation set stopped improving for 3 epochs to allow for fine learning. All the parameters were initialized with the default ImageNet weights. We trained the model for 50 epochs with a batch size of 64 and chose the model with optimal performance on the validation set.
Randomization	To train the DeepQuality, 32112 labeled fundus images were randomly divided into the training set, development set, and internal test set at a ratio of 3:1:1. There was no patient overlap between these sets.

Blinding	Two certified retinal experts with at least five years of clinical experience in pediatric ophthalmology were recruited to label all anonymized images independently. To ensure the reliability of image annotation, reference standards were determined only when both retinal experts reached consensus. All disputed images were submitted to another senior retinal expert with more than ten years of clinical experience for arbitration.

Reporting for specific materials, systems and methods

We require information from authors about some types of materials, experimental systems and methods used in many studies. Here, indicate whether each material, system or method listed is relevant to your study. If you are not sure if a list item applies to your research, read the appropriate section before selecting a response.

Materials & experimental systems

n/a	Involved in the study
☒	☐ Antibodies
☒	☐ Eukaryotic cell lines
☒	☐ Palaeontology and archaeology
☒	☐ Animals and other organisms
☐	☒ Clinical data
☒	☐ Dual use research of concern
☒	☐ Plants

Methods

n/a	Involved in the study
☒	☐ ChIP-seq
☒	☐ Flow cytometry
☒	☐ MRI-based neuroimaging

Clinical data

Policy information about clinical studies
All manuscripts should comply with the ICMJE guidelines for publication of clinical research and a completed CONSORT checklist must be included with all submissions.

Clinical trial registration	The study was approved by the Institutional Review Board of Zhongshan Ophthalmic Center at Sun Yat-sen University (IRB-ZOC-SYSU, ID: 2023KYP1029).
Study protocol	The trial protocol is available from the corresponding author.
Data collection	We retrospectively collected 2056260 infantile fundus photographs from January 2016 to June 2022,in Zhongshan Ophthalmic Center (ZOC), Maternal and Children's Hospital of Liuzhou (LZH), Maternal and Children's Hospital of Linyi (LYH), and Qilu Hospital (QLH).
Outcomes	The primary outcomes were the AUCs of DeepQuality. The secondary outcomes included the accuracy, sensitivity and specificity of the DeepQuality and the clinicians under various settings.
